# Correction: Jiang et al. Methods for Obtaining One Single Larmor Frequency, Either *v*_1_ or *v*_2_, in the Coherent Spin Dynamics of Colloidal Quantum Dots. *Nanomaterials* 2023, *13*, 2006

**DOI:** 10.3390/nano16120707

**Published:** 2026-06-09

**Authors:** Meizhen Jiang, Yuanyuan Zhang, Rongrong Hu, Yumeng Men, Lin Cheng, Pan Liang, Tianqing Jia, Zhenrong Sun, Donghai Feng

**Affiliations:** 1 State Key Laboratory of Precision Spectroscopy, East China Normal University, Shanghai 200241, China; 52200920010@stu.ecnu.edu.cn (M.J.); 52170920029@stu.ecnu.edu.cn (Y.Z.); 52250920010@stu.ecnu.edu.cn (Y.M.); 51210920033@stu.ecnu.edu.cn (L.C.); tqjia@phy.ecnu.edu.cn (T.J.); zrsun@phy.ecnu.edu.cn (Z.S.); 2 College of Sciences, Shanghai Institute of Technology, Shanghai 201418, China; 3 College of Arts and Sciences, Shanghai Dianji University, Shanghai 201306, China; liangp@sdju.edu.cn; 4 Collaborative Innovation Center of Extreme Optics, Shanxi University, Taiyuan 030006, China

In the original publication [1], there was a mistake which affected Figures 4 and 5 as published. The data in Figure 4b (main panel) remain unchanged. In the previously published version, however, the *y*-axis values were divided by 0.5. We have now dropped this scaling factor, so all curves in the main panel are halved. Consequently, the blue curve in Figure 4c—corresponding to the blue curve in Figure 4b—is also halved, whereas the red curve in Figure 4c is left unchanged.

The original data in Figure 5a,b (main panels) also remain unchanged. However, in the previously published version the *y*-axis values were incorrectly scaled by 0.1; they have now been rescaled by 0.015, the same as for Figure 4a. Consequently, all curves in Figure 5a (main panel) are increased by a factor of ~6.67. Likewise, the *y*-axis values in Figure 5b (main panel) have been divided by a factor of 4. We note that there is a discrepancy in the scaling factor labeled in the Figure 5b inset in the previously published version: it states ‘×7’, but the actual scaling factor should be ‘×2.8’. Because 2.8 is a strange number, the data are now multiplied by 3 and the corresponding FFT signal becomes a little stronger. Accordingly, the figure caption in Figure 5b has also been updated.

The corrected Figure 4 and Figure 5 appear below:

**Figure 4:**
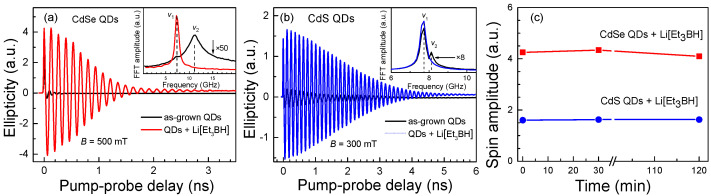
(**a**,**b**) Time-resolved ellipticity signals in as-grown colloidal CdSe and CdS QDs and QDs with Li[Et_3_BH] prepared in a N_2_ atmosphere. The insets show the corresponding FFT spectra of panels a and b. The FFT spectra of as-grown CdSe and CdS QDs are multiplied by 50 and 8, respectively, for better distinction of the two observed Larmor frequencies. (**c**) Spin amplitudes in CdSe and CdS QDs with Li[Et_3_BH] as a function of time under ambient room light illumination. The molar ratios of Li[Et_3_BH] to CdSe and CdS QDs are 500 and 10, respectively. The laser repetition rate is 50 kHz.

**Figure 5:**
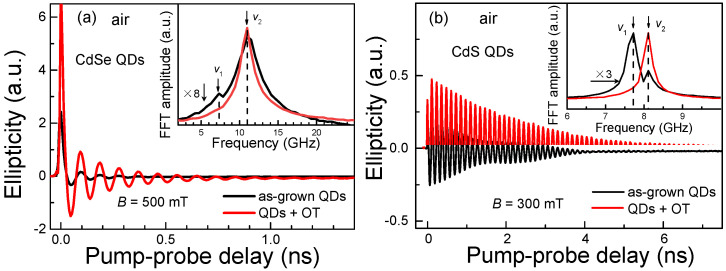
(**a**) Time-resolved ellipticity signals of as-grown colloidal CdSe QDs and QDs with 1-octanethiol (OT) prepared in an air atmosphere. The inset shows the corresponding FFT spectra. The FFT spectroscopy of as-grown CdSe QDs is multiplied by 8 for clarity. The molar ratio of OT to CdSe QDs is 11,000. The laser repetition rate is 50 kHz. (**b**) Spin signals of as-grown colloidal CdS QDs and QDs with OT prepared in an air atmosphere. The inset shows the corresponding FFT spectra. The FFT spectroscopy of as-grown CdS QDs is multiplied by 3 for clarity. The molar ratio of OT to CdS QDs is 5000. The laser repetition rates for as-grown CdS QDs and QDs with OT are 50 and 10 kHz, respectively.

The authors state that the scientific conclusions are unaffected. This correction was approved by the Academic Editor. The original publication has also been updated. 
